# An Insect- and Rain-Proof Net Raises the Production and Quality of Chinese Bayberry by Preventing Damage From Insects and Altering Bacterial Communities

**DOI:** 10.3389/fpls.2021.732012

**Published:** 2021-09-16

**Authors:** Haiyue Yu, Shengke Tian, Qianbin Huang, Jiuzhou Chen, Yuping Wu, Runze Wang, Lingli Lu

**Affiliations:** ^1^MOE Key Laboratory of Environment Remediation and Ecological Health, College of Environmental and Resource Science, Zhejiang University, Hangzhou, China; ^2^Zhejiang Provincial Key Laboratory of Subtropic Soil and Plant Nutrition, Zhejiang University, Hangzhou, China; ^3^Huangyan Agricultural and Rural Bureau, Taizhou, China; ^4^Ningbo Agricultural and Rural Bureau, Ningbo, China

**Keywords:** Chinese bayberry, fruit quality, sugar, organic acid, bacterial community

## Abstract

Chinese bayberry (*Myrica rubra*) is a popular, nutrient- and antioxidant-rich fruit in Asia. However, it is susceptible to *Drosophila* during ripening, which disrupts production and causes economic loss. This study compared the effects of insecticides, insect-proof nets (IPNs), and insect- and rain-proof nets (IRPNs) on Chinese bayberry production and quality. *Drosophila* was absent in fruits from IPN- or IRPN-treated trees but only significantly reduced by insecticides. IPNs and IRPNs significantly increased fruit diameter, weight, edible rate and the Brix/acid ratio, and IRPNs had the strongest effect. Analysis of 16S rDNA showed that fruits collected from differently treated trees had unique bacterial communities. In IRPN fruits, *Acetobacter* and *Gluconobacter* were significantly decreased, reducing sugar consumption and disease; in addition, PICRUSt analysis predicted imputed functional profiles related to carbohydrate and nitrogen metabolism and mineral transport for fruit growth and development. This study proposed the use of IRPNs for improving Chinese bayberry production and quality.

## Introduction

Chinese bayberry (*Myrica rubra* Sieb. et Zucc.) is a subtropical fruit tree native to China and other Asian countries, and its cultivation has been recorded in Chinese history for more than 2,000years (Sun, et al., 2013). Bayberry fruit is delicious with an attractive colour and flavour, and contains high levels of cyanidin-3-glucoside, minerals and antioxidants, which exhibit bioactivities that counteract inflammation, allergens, diabetes, cancer, bacterial infection and diarrhoea (Yang et al., 2011; Sun et al., 2012; Huang et al., 2014). Bayberry fruit is of high economic value and can be eaten or processed into products, such as canned syrup, candied fruit, fruit juice, jam and fruit wine (Cao et al., 2020; Fang et al., 2020). The Dong Kui cultivar is one of the main varieties of Chinese bayberry cultivated in Southeast China and is highly favoured by consumers because of its large size, good quality, and sweet and unique flavour (Cheng et al., 2016; Yu et al., 2020). Chinese bayberry (Dong Kui cultivar) fruits contain abundant soluble sugars, organic acids, cellulose, minerals, vitamins, fibre, phenolics and amino acids that are beneficial to the human body (Shi et al., 2014).

During the maturation stage, Chinese bayberry fruits are highly susceptible to pests, especially *Drosophila suzukii* (Liu et al., 2018), which is a serious economic pest of stone and small fruits because the female lays eggs within ripening fruits before harvest, leading to massive agricultural damage and severe production losses (Goodhue et al., 2011; Lee et al., 2011a). The conventional method of killing Chinese bayberry fruit pests, including *Drosophila*, is to use insecticides, but pests within the fruit flesh (mainly *Drosophila* larvae) are difficult to exterminate by spraying with insecticides (Atallah et al., 2014; Sparks et al., 2020). Moreover, high levels of insecticide residues on the fruits, trees and in the soil may cause hidden agricultural and environmental damage, and pose a danger to human health (Lee et al., 2011b; Fenner et al., 2013). Physical and chemical measures, such as light traps, sticky traps, sex attractants and poison baits, are also not ideal for *Drosophila* control because the adults do not have a strong tropism (Burrack et al., 2015). Insect-proof nets (IPNs) are a purely physical approach to separate trees from pests and thus can effectively minimise their infection by insect pests (Guo et al., 2015). Although not scientifically reported, IPNs are widely used on Chinese bayberry trees in fields and significantly increase the fruit production and economic benefits of the trees. It is clear that using a single IPN to cover each tree provides a physical barrier to reduce the migration of *Drosophila* adults into the orchard, while the direct and indirect effects of IPNs on the quality of Chinese bayberry fruits and its mechanisms remain unclear.

In addition to *Drosophila*, excessive rain flow during the harvesting season of Chinese bayberry is another important factor leading to serious reductions in fruit yield. The Chinese bayberry blooms in April, and the fruits mature from June to July, which coincides with the rainy season in Southeast China. High humidity generally causes a serious reduction in bayberry fruit before harvest and decreases its storage quality. Rain-proof cultivation techniques can prevent fruit trees from being exposed to excessive rain during the maturation period to avoid losses due to fruit dropping off the trees, as has been reported for grapes, berries, cherries and mangoes (Li et al., 2014; Suran et al., 2019; Xu et al., 2019; Dai et al., 2020). However, it is difficult and costly to undertake the large-scale construction of overall rain-proof films for Chinese bayberry trees since they are generally planted on hillsides with complex terrain.

In this study, we report an economical and feasible rain-proof technology involving directly covering the crown of a Chinese bayberry tree with an IPN and plastic rain film during its ripening stage. The effects of preventing insect infestations and avoiding rain on the production and quality of Chinese bayberry fruit were investigated using the Dong Kui cultivar grown in a natural environment with different treatments, including insecticide, IPN, and insect- and rain-proof net (IRPN). High-throughput sequencing of 16S rRNA gene amplicons was performed to characterise the microbiome associated with the Chinese bayberry fruits under the different treatments to understand possible interactions of the bacterial community with insect protection and rain proofing, and their effects on fruit production and quality.

## Materials and Methods

### Plant Growth and Treatments

Chinese bayberry trees were grown under the natural environment (controls) or treated separately with insecticides, IPNs or IRPNs. An insecticide solution containing 0.44% tetramethrin, permethrin and cypermethrin (1:1:1) was used to trap and kill *Drosophila* between the hard-core and mature stages of the Chinese bayberry. IPN-treated Chinese bayberry trees were covered with IPNs 1month before harvest to prevent *Drosophila* from coming into contact with the Chinese bayberries (Bai et al., 2020), and IRPN-treated Chinese bayberry trees were covered with rain-proof films and IPNs to prevent contact with both *Drosophila* and rainwater.

Mature Chinese bayberry fruits were collected from the differently treated trees, in Taizhou, Zhejiang Province, China, on 2 July 2020, packaged in ice bags, and transported to the laboratory, where they were preserved at 4°C until used for subsequent analysis. The selection criteria for the types of trees were the same species of Chinese bayberry (Dong Kui cultivar), 15years old, with a tronc diameter of 20cm; the vitality was strong, and the yield was close before treatments. The trees were planted in the same area to minimise the impact of environmental factors, such as soil, precipitation, light and temperature on the experimental results. The trees between the different treatments were relatively close, and they are basically planted on the same piece of soil. Especially, between the control and the net cover treatments, the trees were only a few metres apart.

### Number of *Drosophila*

Thirty Chinese bayberries at similar stages of maturity were randomly selected, packed in a fresh-keeping bag and stored at room temperature (25°C) for 7days. After all the *Drosophila* on the Chinese bayberries had pupated, the number of *Drosophila* larvae on each bayberry was counted.

### Fruit Quality

A total of 50 fresh Chinese bayberries from trees in each treatment group were used for the analyses of lateral particle size with Vernier callipers and fruit weight. Fresh Chinese bayberries were separated into pulp and kernel and weighed to calculate the edible ratio. The edible parts (pulp) of the berries were washed with deionised water, blended in a juicer and filtered through a 400-mesh filter cloth. The soluble solids content (SSC) in the Chinese bayberry juice was measured using a pocket refractometer (ATAGO Co., Ltd., Tokyo, Japan) with an accuracy of 0.001 Brix (Yuan et al., 2020). The titratable acidity content (TAC) of Chinese bayberry juice samples was titrated by adding 1.0M sodium hydroxide (NaOH) and phenolphthalein (Lobit et al., 2002). The Brix/acid ratio was calculated by SSC/TAC.

### Soluble Sugars

The Chinese bayberry pulp was freeze-dried in liquid nitrogen and ground into powder (stored in a freezer at −80°C). To 1.0g (accurate to 0.000lg) of frozen sample powder, 5ml of 80% ethanol was added; after 30min in a constant temperature water bath at 80°C, the sample was centrifuged at 10000rpm for 15min. The supernatant was removed, 5ml of 80% ethanol was added to the residue, and the extraction was repeated. The extracted supernatants were combined, and the ethanol was evaporated in a water bath at 90°C, and deionised water was added to bring the volume to 5ml (Ma et al., 2015).

After filtration through a 0.22-μm microporous membrane, the sugar content in the pulp was analysed by high performance liquid chromatography (HPLC; Agilent 1,200 LC, Agilent, Santa Clara, CA, United States) with a refractive index detector; three samples were analysed for each treatment. The Polaris NH2 column (4.6×250mm, 5μm; Agilent) was used; the mobile phase was acetonitrile:water (4,1, v:v) containing 0.1% ammonia, the flow rate was 1.2ml/min, the injection volume was 20μl, and the temperature was 30°C (Wang et al., 2016).

### Organic Acids

1gram (accurate to 0.000lg) of frozen sample powder was homogenised in 5ml of sodium dihydrogen phosphate buffer (pH=3) and incubated for 30min at 4°C. After extraction, the homogenate was centrifuged at 10,000rpm for 15min at 4°C. The above extraction steps were repeated once more, and the supernatants were combined. After filtration through a 0.22-μm microporous membrane, the organic acid content in the pulp was analysed by HPLC (Agilent 1200LC; Agilent) with a diode array detector (DAD); three samples were analysed for each treatment. The ZORBAX SB-Aq column (4.6×250mm, 5μm; Agilent) was used; the mobile phase was sodium dihydrogen phosphate buffer (pH=3), the flow rate was 0.5ml/min, the injection volume was 10μl, and the temperature was 25°C (Xu et al., 2015).

### Nutrient Elements

Chinese bayberry pulp was dried in an oven at 65°C to a constant weight, and 5g of the dried sample was digested with concentrated nitric acid (HNO_3_) in a digestion oven at 180°C until it cleared. An appropriate amount of hydrogen peroxide (H_2_O_2_) was added to continue digestion until the solution was transparent. The solution was cooled to room temperature, diluted with an appropriate amount of deionised water, and filtered through a 0.22-μm microporous membrane; the content of inorganic nutrient elements was measured by inductively coupled plasma–atomic emission spectrometry.

The index of nutritional quality (INQ) was used for quantitative and qualitative analysis of single nutrients; INQ is the ratio of the reference intake of each nutrient to the average requirement of energy provided by the food (Palacin-Arce et al., 2015; Alireza et al., 2020). The INQ of K, Ca, Mg, Fe, and Zn for bayberry fruits that had undergone different treatments was calculated according to a previous report as follows (CNS, 2014): *INQ = (the content of a certain nutrient in a certain food/recommended intake of this nutrient)/(energy provided by this certain food/recommended intake of energy)*.

### Microbiological Analysis

The 16S rDNA gene sequences of the microorganisms found in fresh Chinese bayberry fruits were determined according to Hou et al. (2017). Briefly, bayberry fruits were submerged in sterilised phosphate-buffered saline solution, sonicated for 1min, and vortexed for 30s. The fruit solutions were filtered through a sterilised membrane and homogenised for DNA extraction using the Plant DNA Extraction Mini Kit B (MoBio, United States). DNA extracts were quantified using a NanoDrop One spectrophotometer (Thermo Fisher Scientific, Waltham, MA, United States). Fragments of the bacterial 16S rRNA gene (V3–V4 region) were amplified using the primer set 338F: 5′-ACTCCTACGGGAGGCAGCA-3′ and 806R: 5′-GGACTACHVGGGTWTCTAAT-3′. An aliquot of 60ng of purified DNA template from each sample was amplified in a total volume of 50μl under the following conditions: 5min at 94°C for initialisation, 30cycles of 30s denaturation at 94°C, 30s annealing at 52°C, and 30s extension at 72°C, followed by a final elongation at 72°C for 10min. Equimolar quantities of PCR products for each sample were combined and sequenced on an Illumina NovaSeq 6,000 platform (Illumina, San Diego, CA, United States) by MeiGe Genomic Biotech (Guangzhou, China). Sequences were analysed using the QIIME 2 pipeline (version 2020.2.0). All sequence data were submitted to the Sequence Read Archive database (NCBI, Bethesda, MD, United States) under bioproject number PRJNA723673.^1^ QIIME 2 was used to determine the taxonomic and phylogenetic α- and β-diversity indices. Taxonomic- and phylogenetic-based β-diversity indices were estimated based on Bray–Curtis distances. Linear discriminant analysis (LDA) was used to determine the significantly discriminant taxa in fruit from trees that had undergone each treatment (using one-against-all comparisons) using the factorial Kruskal–Wallis sum-rank test (*α*=0.05). Discriminant taxa were used to generate taxonomic cladograms. PICRUSt2 (version 2.4.1) was used to predict the functional potential of microbial communities (Douglas et al., 2020).

### Statistical Analysis

All data from the characterisation of the Chinese bayberries are reported as means±standard deviations. The figures were plotted using the Origin software (version 8.0; OriginLab Corp., Northampton, MA, United States), and one-way analysis of variance (ANOVA) was performed using SPSS software (version 21.0; IBM Corp., Armonk, NY, United States). Significant differences were detected by Duncan’s test (*p*<0.05).

## Results

### *Drosophila* Growth on Chinese Bayberry Fruits

**Figure 1:**
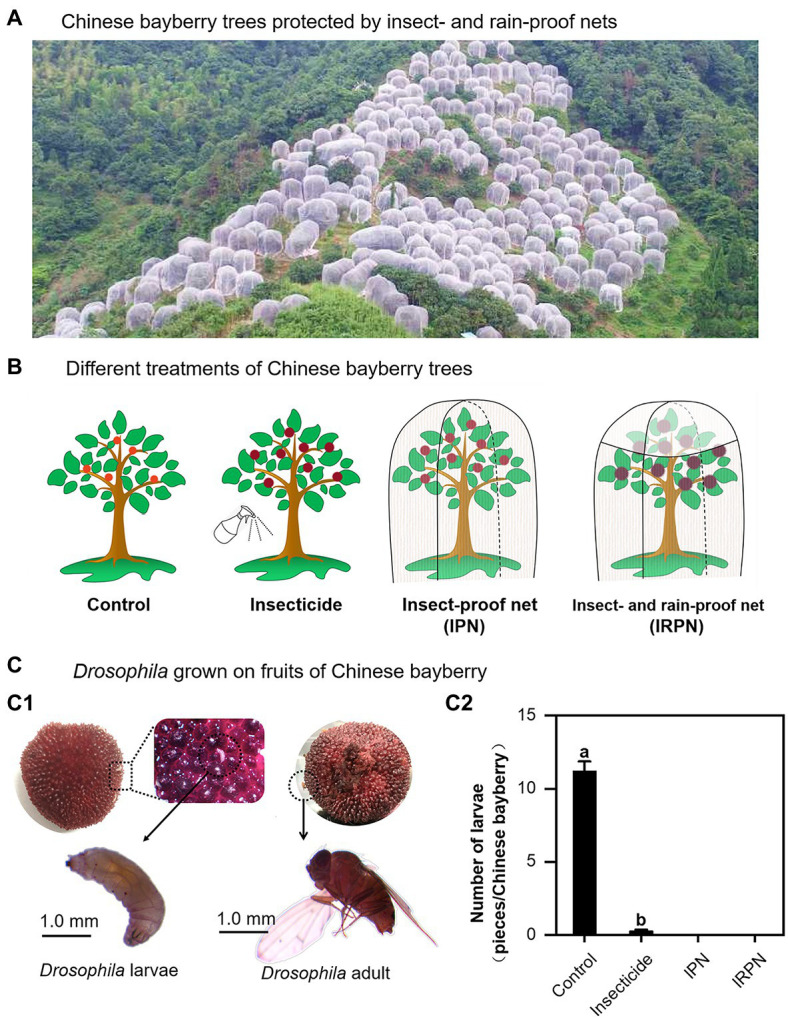
*Drosophila* growth on Chinese bayberry fruits collected from trees grown in different environments. **(A)** Field image of Chinese bayberry trees protected by insect-proof nets (IPNs) and rain-proof films during the fruit maturation period. **(B)** The trees were grown under natural conditions (controls) or treated separately with insecticides, IPNs or insect- and rain-proof nets (IRPNs). **(C)** *Drosophila* (C1) and number of *Drosophila* (C2) on Chinese bayberry fruits collected from untreated and treated trees.

Covering the crown of a Chinese bayberry tree with an IRPN during ripening successfully prevented pest infestation and rain flow, even for those planted on a hillside with complex terrain (Figure 1A). The trees in the field were either grown in the natural environment (controls) or treated separately with insecticide, IPNs or IRPNs (Figure 1B). The main pests on the surface of Chinese bayberry fruits were *Drosophila*, and more than 10 *Drosophila* larvae were observed on a single bayberry fruit collected from a control tree (Figure 1C). Compared with the controls, the numbers of *Drosophila* larvae and adults were significantly, but not completely, decreased in bayberry fruits collected from trees sprayed with insecticides. Interestingly, application of a single IPN completely prevented infestation of bayberry fruits by *Drosophila*, and the addition of an IRPN provided similar protection against *Drosophila* (Figure 1C).

### Fruit Yield of Chinese Bayberry Trees

**Figure 2:**
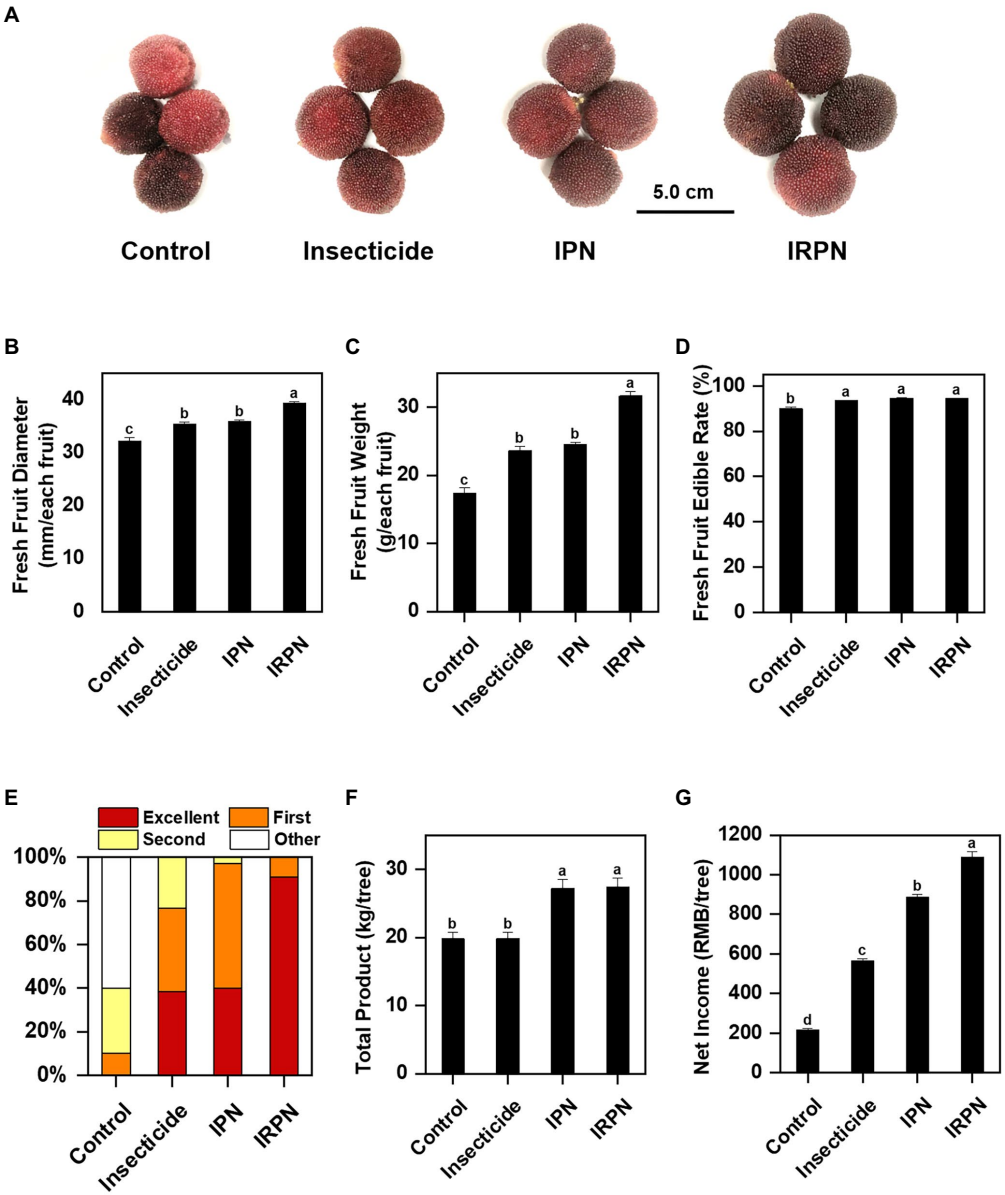
Fruit yields of Chinese bayberries grown under different planting conditions. The trees were grown under natural conditions (controls) or treated separately with insecticides, IPNs or IRPNs. **(A)** Photos of fresh Chinese bayberries. (B–D) Fruit diameter (mm/each fruit) **(B)**, weight (g/each fruit) **(C)** and edible rate (%) **(D)** measured in a single Chinese bayberry. **(E)** Percentages of Chinese bayberry fruits of different grades according to commercial standards (special ≥25g, 25g > first ≥21g, 21g> second ≥18g, 18g>other). **(F)** Total fruit yield (kg/tree) of each Chinese bayberry tree. (G) Total net income (RMB/tree) from each Chinese bayberry tree.

Analysis of the diameter, weight and total yield of fruits collected from Chinese bayberry trees undergoing different treatments showed that insect protection and rain proofing significantly improved the production and quality of the bayberry fruits (Figure 2). Compared with the controls, both the diameters and weights of the fruits increased significantly when the trees were treated with insecticides or IPNs, but the increases were most pronounced when the plants were protected from both insects and rain by IRPNs. The IRPN resulted in incremental increases in fruit diameter and weight of +22.6% and +82.4%, respectively, compared with the controls (Figures 2B,C). The application of IRPNs significantly improved the yield and economic value of the Chinese bayberry trees. As shown in Figure 2E, more than 91.0% of the mature bayberries could be classified as excellent special grade. The total fruit yield and net income from each Chinese bayberry tree treated with an IRPN increased by 1.4- and 5.1-fold, respectively, compared with the controls (Figures 2F,G).

### Sugar and Organic Acids in Chinese Bayberry Fruits

**Figure 3:**
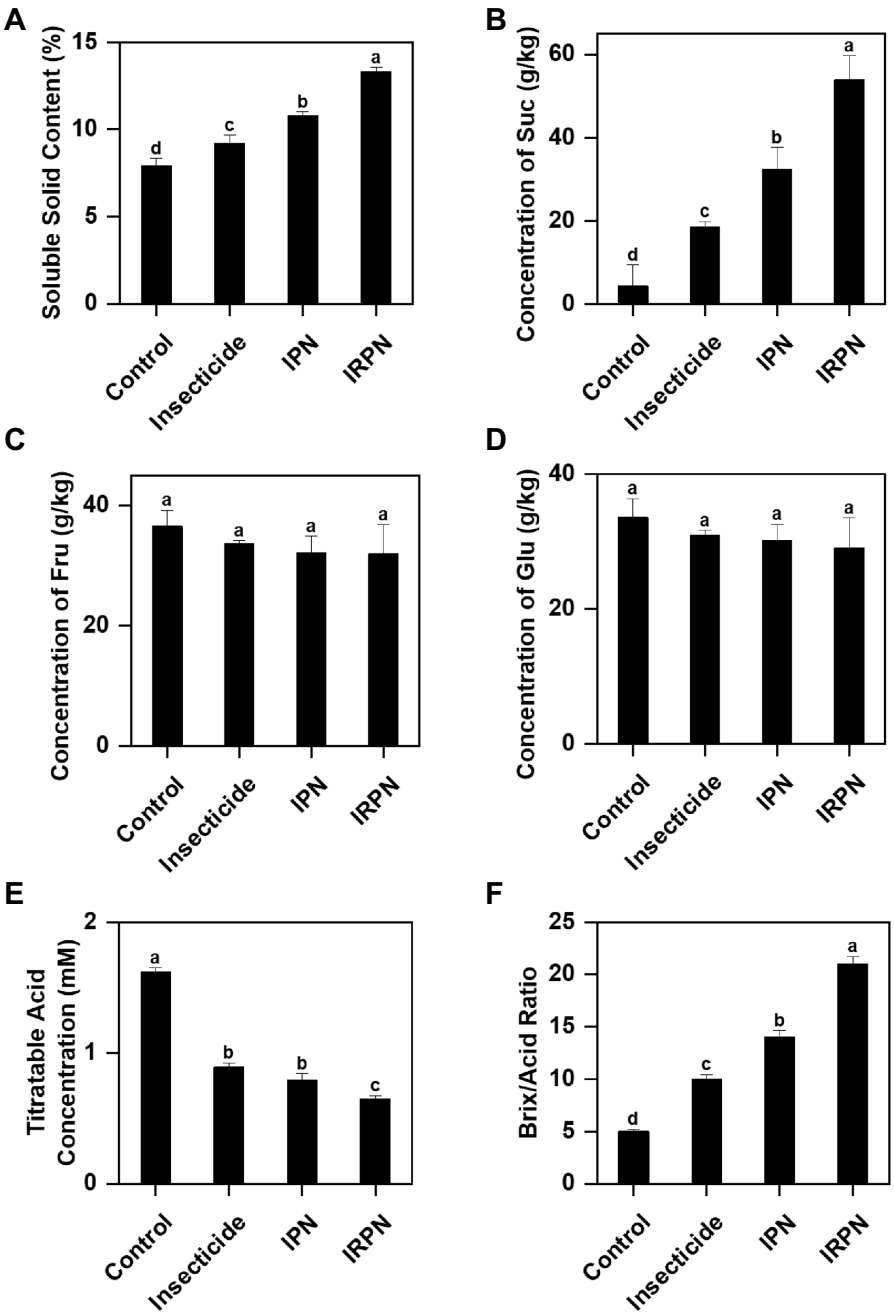
Contents of sugars and organic acids in Chinese bayberry grown under different conditions. Soluble solid content (%) **(A)**, sucrose (g/kg) **(B)**, fructose (g/kg) **(C)**, glucose (g/kg) **(D)**, titratable acid (mM) **(E)** and the Brix/Acid ratio **(F)** were analysed for fruit juice of Chinese bayberry collected from trees grown under natural conditions (controls) or treated separately with insecticides, IPNs or IRPNs.

Protection against insects and rain significantly improves the accumulation of sugar and soluble solids in Chinese bayberry fruits. Compared with the controls, the soluble solid contents of fruits showed significant increases of 16.5%, 36.7% and 68.4% under insecticide, IPN and IRPN treatments, respectively (Figure 3A). Treatments with IPNs or IRPNs significantly improved the sucrose concentration of Chinese bayberry fruits, and the effects were most pronounced when trees were protected from both insects and rain by IRPNs. The sucrose concentration of bayberry fruits from the trees treated with IRPNs was 12.8 times higher than that in the control fruits (Figure 3B), even though the sizes and weights of IRPN-treated bayberries were considerably higher than those of the controls (Figure 2A). The fructose and glucose concentrations of the bayberry fruits collected from trees undergoing different treatments were not significantly different (Figures 3C,D).

The opposite trend was observed for titratable acid in fruits from trees undergoing different treatments. A significant reduction in acid content was observed in fruits treated with insecticides (−45.1%), IPNs (−51.2%) and IRPNs (−59.9%) compared with the controls (Figure 3E). As a result, the Brix/acid ratios in fruits from trees undergoing all three treatments were significantly higher than those of the controls, and the effect was most pronounced in fruits from trees grown under IRPNs (Figure 3F). Analysis of organic acids showed that the concentrations of citric acid, which is the most abundant organic acid in bayberry fruits, significantly decreased under insecticide, IPN and IRPN treatments (Table 1). The citric acid content in fruits from IRPN-treated trees was only 5.60±0.20g/kg, which was less than one-third that in the control fruits (17.03±0.30g/kg). In contrast, the concentrations of malic acid were 6.12-, 3.46- and 5.20 times greater in fruits from trees undergoing insecticide, IPN and IRPN treatments, respectively, compared to the control fruits (Table 1). No significant variation was observed for oxalic acid, tartaric acid and ascorbic acid in bayberry fruits from trees undergoing different treatments (Table 1).

**Table 1:** Concentration of organic acids in Chinese bayberry in different planting environments.

Treatments^*^	Citric acid (g/kg)	Malic acid (mg/kg)	Oxalic acid (mg/kg)	Tartaric acid (mg/kg)	Ascorbic acid (mg/kg)
Control	17.03 ± 0.30^a^	223 ± 20.3^d^	231 ± 15.4^b^	115 ± 6.7^b^	4.96 ± 1.00^a^
Insecticide	10.15 ± 0.06^b^	1,366 ± 17.6^a^	376 ± 15.0^a^	139 ± 7.5^b^	9.88 ± 2.10^a^
IPN	8.05 ± 0.45^c^	773 ± 31.8^c^	201 ± 8.1^b^	123 ± 3.4^b^	8.00 ± 2.25^a^
IRPN	5.60 ± 0.20^d^	1,162 ± 18.6^b^	207 ± 5.7^b^	182 ± 16.5^a^	5.52 ± 1.53^a^

IPN, insect-proof net; IRPN, insect- and rain-proof net.

* The mean and standard deviation (*n*=3) were calculated for three replicates. The values in a column with different superscript letters a, b, c and d are significantly different (*p*<0.05).

### Mineral Nutrients of Chinese Bayberry Fruits

The total accumulation and concentrations of mineral nutrients were analysed in bayberry fruits grown on trees subjected to different treatments. It is clear that the total accumulation of all the minerals within a single bayberry was much higher in fruit grown in trees treated with insecticides, IPN or IRPN than in the control fruits (Table 2). Due to the large size and biomass of the fruits collected from the trees treated with insecticides, IPN or IRPN (Figure 2), the concentrations of all analysed minerals (including K, Ca, Mg, Fe and Zn) were significantly reduced in these fruits compared with those of the controls (Supplementary Table S1). Nevertheless, the INQs of all bayberry fruits were higher than 1, regardless of the treatment (Supplementary Table S1).

**Table 2:** Nutrient element content of a single Chinese bayberry from trees grown in different planting environments.

Treatments	Mineral nutrients^*^				
	K	Ca	Mg	Fe	Zn
Control	11.3 ± 1.57^c^	1.41 ± 0.06^a^	0.87 ± 0.12^c^	81 ± 13.7^b^	22.0 ± 2.14^b^
Insecticide	21.9 ± 0.62^b^	1.19 ± 0.25^a^	1.41 ± 0.10^b^	94 ± 11.0^ab^	25.4 ± 2.95^b^
IPN	20.8 ± 0.97^b^	1.35 ± 0.12^a^	1.89 ± 0.09^a^	102 ± 10.6^ab^	29.0 ± 1.80^b^
IRPN	25.9 ± 1.06^a^	1.44 ± 0.18^a^	1.99 ± 0.16^a^	124 ± 16.7^a^	53.7 ± 4.03^a^

IPN, insect-proof net; IRPN, insect- and rain-proof net.

* The units for each index were as follows: K (mg/fruit), Ca (mg/fruit), Mg (mg/fruit), Fe (μg/fruit) and Zn (μg/fruit). The mean and standard deviation (*n*=3) were calculated for three replicates. The values in a column with different superscript letters a, b and c are significantly different (*p<*0.05).

### Bacterial Communities of Chinese Bayberry Fruits

**Figure 4:**
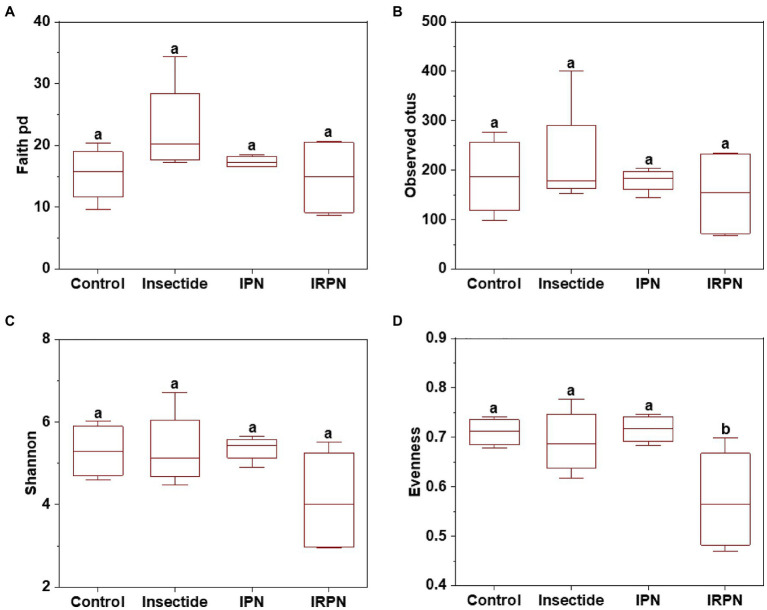
α-diversity of microbial communities of Chinese bayberry trees grown under different treatments. Faith_pd **(A)**, observed_otus **(B)**, Shannon **(C)** and evenness **(D)** of the bacterial communities in fruits collected from trees grown under natural conditions (controls) or treated separately with insecticides, IPNs or IRPNs.

**Figure 5:**
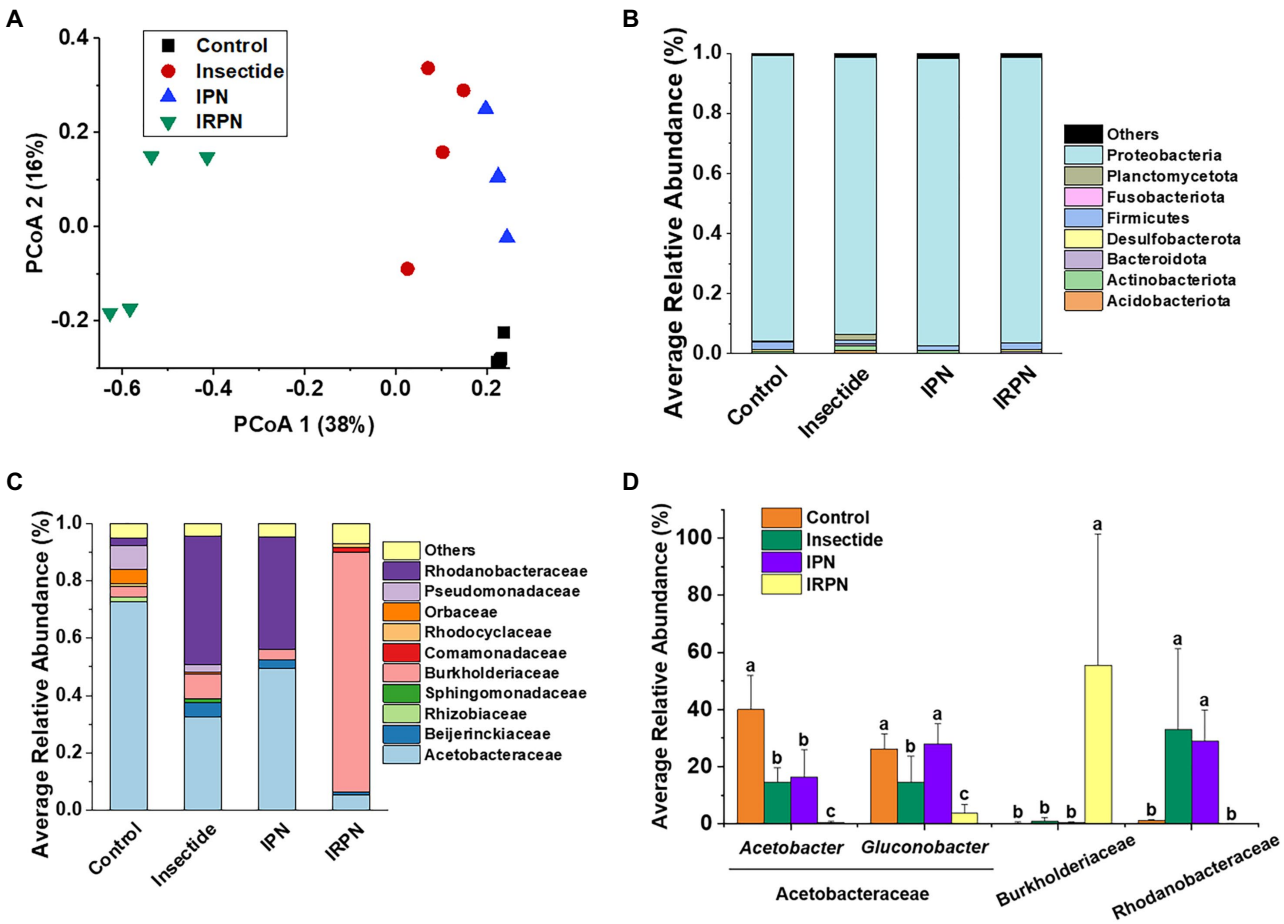
Distribution of the dominant taxa of the bacterial communities. **(A)** Principal coordinate analysis (PCoA) plots for visualising the unweighted UniFrac distance among the bacterial communities. **(B)** Relative abundances of bacterial phyla (%) (phylum level for bacteria) and **(C)** distribution of families (%) affiliated with the phylum Proteobacteria in the Chinese bayberry trees that underwent different treatments. **(D)** Average abundances of key genera (phylum Proteobacteria) in Chinese bayberry grown in different planting environments (%).

The index of evenness and PCoA of microbial community diversity in fruits were significantly affected by IRPN treatment compared with the other treatments, whereas the observed_otus, faith_pd and Shannon indices of microbial diversity in fruits were not significantly different between treatments (Figures 4, 5A).

**Figure 6:**
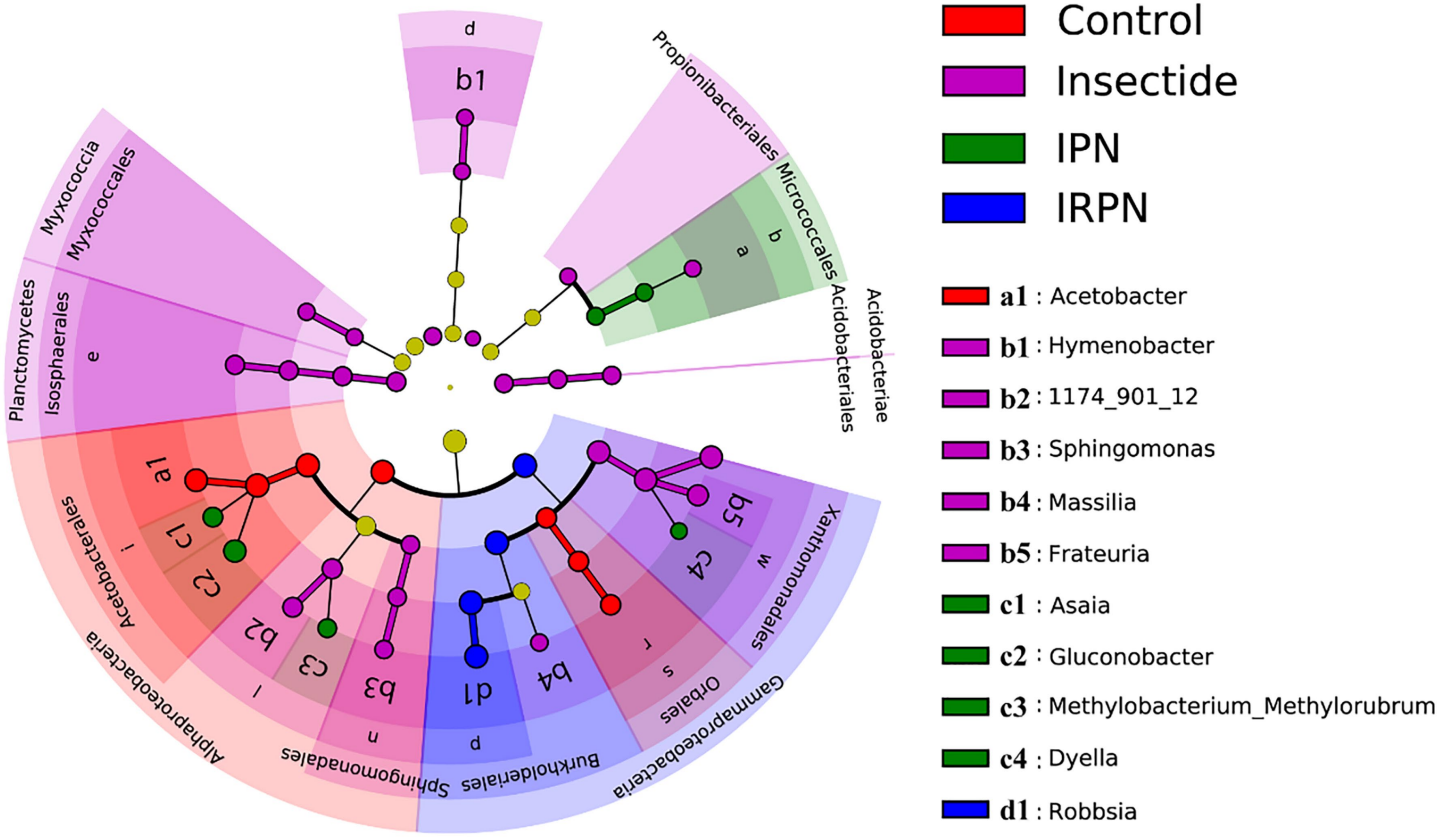
Least discriminant analysis (LDA) effect size taxonomic cladogram for comparing samples collected from the Chinese bayberry trees grown in different planting environments. The discriminant taxon nodes are coloured, and branches are shaded according to the highest ranked group for that taxon. If the taxa were not significantly different between sample groups, the corresponding node is coloured yellow. Select highly abundant taxa are denoted with letters.

The most abundant bacterial operational taxonomic units (OTUs) for all the fruit samples were Proteobacteria, Firmicutes, Bacteroidota, Desulfobacterota, Actinobacteriota and Acidobacteriota, of which Proteobacteria represented the core microbiome of the Chinese bayberry regardless of treatment (Figure 5B). Interestingly, the application of IRPNs resulted in reductions of 3.3%, 3.5% and 31.6% for Proteobacteria, Firmicutes and Desulfobacterota, respectively, and an incremental increase of 2.2 times for other phylum compared with the controls (Figure 5B). When Proteobacteria were classified at the family and genus levels, it was found that the IRPN planting environment selectively reduced the relative abundances of Acetobacteraceae (genera *Acetobacter* and *Gluconobacter*), Rhodanobacteraceae (genus *Frateuria*) and Orbaceae in the bayberry fruits compared with the other treatments (Figures 5C,D). However, a significantly elevated relative abundance of Burkholderiaceae was observed in the fruits of Chinese bayberry grown with IRPNs (Figures 5C,D). The LDA effect size taxonomic cladogram confirmed that there were significant differences in bacterial communities in fruit from trees undergoing different treatments, and IRPN application gave rise to a distinct bacterial community that selectively attracted certain bacteria, especially Burkholderiaceae (Figure 6).

**Figure 7:**
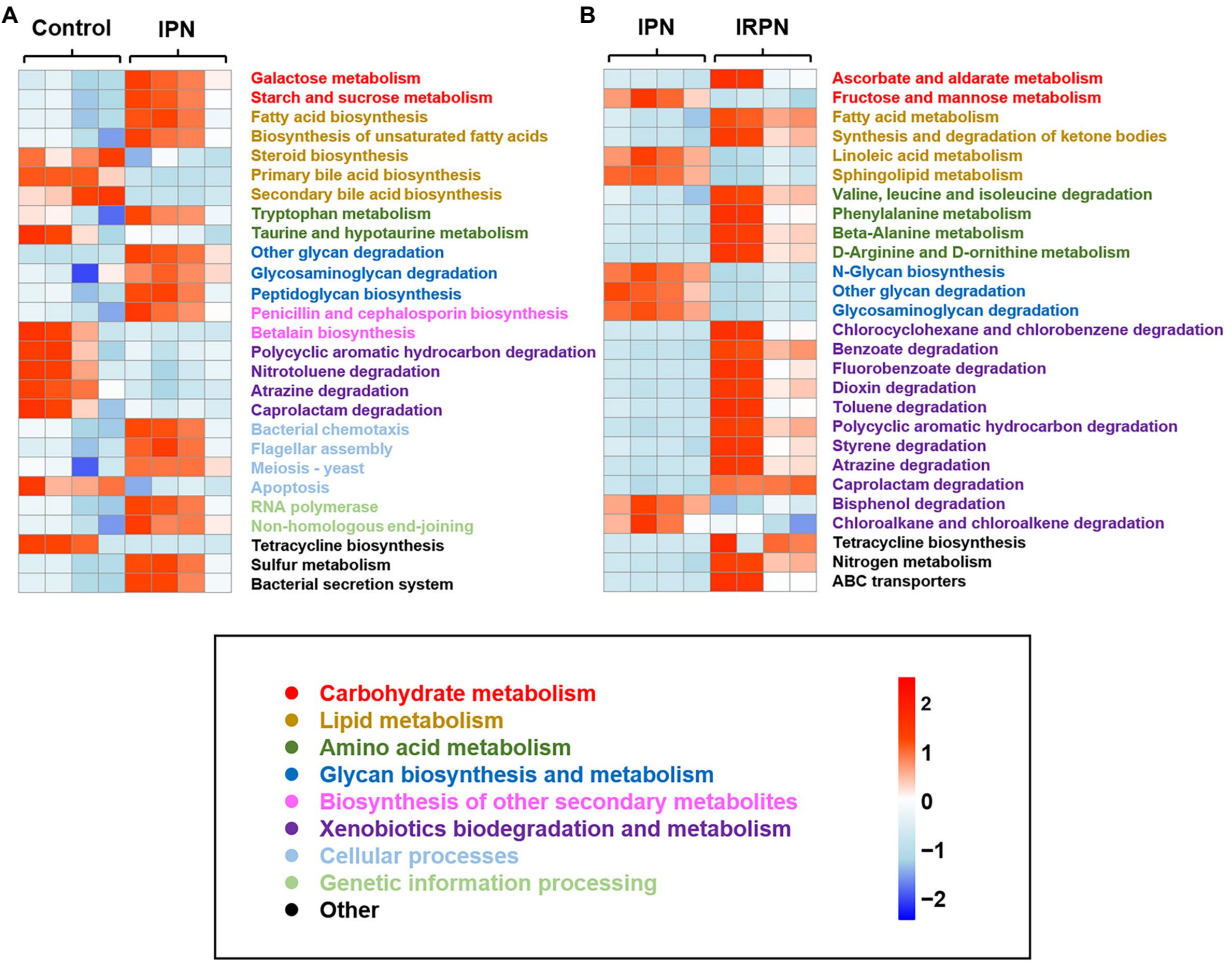
Heat map of the relative abundance of key functional gene families predicted by PICRUSt of control and IPN **(A)** and IPN and IRPN **(B)**. Gene families are coloured according to functional category.

PICRUSt is a bioinformatics tool used to predict metagenome functional content. The relative abundances of key functional gene families predicted by PICRUSt suggested higher relative abundances of imputed functional profiles related to carbohydrate and lipid metabolism in bayberry fruits after application of IPN or IRPN compared with the controls (Figure 7). Interestingly, the prevention of insects by application of IPN significantly altered the bacterial chemotactic and secretion systems in bayberry fruits (Figure 7A). Compared with IPN, protection from both insects and rain by use of IRPNs on the bayberry trees significantly increased the fruits’ functional profiles associated with amino acid metabolism, as well as biodegradation of xenobiotics, including chlorocylohexane, benzoate, dioxin, toluene, polycyclic aromatics, styrene, atrazine and caprotactam (Figure 7B). IRPN treatment also triggered higher levels of expression of predicted ATP-binding cassette (ABC) gene families and nitrogen metabolism genes in bayberry fruits (Figure 7B).

## Discussion

Chinese bayberry is very sensitive to insects (especially *Drosophila*) and pathogens during both ripening and storage because its edible part is the first outside layer and rich in sugars, nutrients and water. However, traditional strategies to protect the fruits by using insecticides may pose residual risks to the fruit, the environment and human health (Devine and Furlong, 2007); in addition, application of insecticides may not completely eliminate the insects, possibly due to the development of insect resistance (Sparks et al., 2020), as confirmed by the results of our study (Figure 1C). IPNs are suitable tools for insect control (Formisano et al., 2020a) and have been widely used on vegetables, melons and fruits (Chien and Chang, 2019; Formisano et al., 2020b). However, the effects of IPNs on Chinese bayberry fruits have not yet been reported. This study suggests that a simple IRPN can be an ideal strategy to improve the fruit production and quality of Chinese bayberry, particularly for those grown naturally on a hillside. The positive effects of IRPNs are due to not only their effective protection of bayberry fruits from *Drosophila* but also their distinct bacterial communities formed by avoidance of insects and excessive rain flow.

The beneficial effects of IRPNs are indicated by the improved fruit yield and nutritional value of Chinese bayberry. It is clear that the protection of bayberry trees from both insects and rain flow significantly enhanced the cell growth of the bayberry fruits, which resulted in extremely large sizes and high weights of the fruits. The commercial value of Chinese bayberry is largely dependent on its fruit size and weight (Ozer et al., 1995; Fu et al., 2016). According to suggested grading standards (LY., 2018), protection from *Drosophila* by using insecticides or IPNs improved the percentage of excellent grade bayberry fruits to 38.5 and 40.0%, respectively, while the excellent grade of IRPN-treated fruits reached as high as 90.9%. Application of IRPNs resulted in 1.4- and 5.1-fold higher fruit yields and net incomes for each single Chinese bayberry tree, respectively, compared with the controls. This suggests that using IRPNs during the fruit ripening stage is an ideal strategy for improving the agricultural and commercial value of Chinese bayberry trees without high environmental costs.

Moreover, IRPNs improved the flavour and nutritional value of Chinese bayberry fruits, which are largely associated with the contents of sugars, amino acids and organic acids in fruits (Shi et al., 2014; Feng et al., 2020). Highly soluble solid content (mainly composed of soluble sugars such as sucrose, glucose and fructose) and low Brix/acid ratios in fruit juice are typical indices that reflect the sweetness and sourness of fruits (Gomez et al., 2002; Jayasena and Cameron, 2008). The higher levels of soluble solids and Brix/acid ratios in the IRPN-treated fruits suggest that they have better flavour and are sweeter than the controls. Consistent with a previous study (Zhang et al., 2015), the main sugar and organic acid components of Chinese bayberry are sucrose and citric acid, respectively. The total accumulation of fructose and glucose also should be significantly increased in bayberry fruits from the IRPN-treated trees because of their significantly increased biomass. The positive effects of IRPN on Chinese bayberry fruit flavour and sweetness are mainly due to the significantly higher sucrose and lower citric acid contents in these fruits than in the controls, as well as their higher free amino acid content. This implies that IRPN treatment enhances carbohydrate and nitrogen metabolism to produce more sucrose and amino acids but less citric acid in bayberry fruits, as discussed below.

Chinese bayberry is loved by consumers because of not only its unique taste but also its richness in mineral nutrients. Fruit is a good food that provides essential mineral nutrients (Gupta and Gupta, 2014; Kumari et al., 2017), but the intake of high sugar from fruits may be associated with obesity (Weichselbaum, 2008; Tappy and Lê, 2010). It is therefore suggested that the INQs of minerals in fruits should be ≥1 for optimal human health, which means that the mineral nutrients inside a food are equal to or higher than the energy (sugar) provided, and the food is of quality nutritional value, especially for people who are overweight and obese. The total accumulation of these minerals significantly increased in each bayberry fruit collected from the trees grown under the IRPNs compared with the controls, with the INQs for all the mineral elements being >1. Therefore, these bayberry fruits can provide adequate mineral nutrients for human health in comparison with the energy they provide.

The positive effects of IRPN use on fruit yield and the nutritional value of Chinese bayberry are due to not only the reduction of *Drosophila* infestations but also its unique bacterial communities, which are formed when the trees avoid both insects and excessive rain. It is previously reported that the microbial community on surface of fruits is greatly affected by the planting environment factors including rain flow (El Sheikha et al., 2009, 2012). The propagation speed of disease-causing microorganisms in fruits increases with the increase of precipitation intensity (Madden et al., 1996), while rain shelter during the fruit development decreased fruit disease (Lim et al., 2015). The Chinese bayberry trees during the development and maturity stage were subjected to both excessive rainfall and *Drosophila*, which might shape the specific microflora on surface of the bayberry fruits. The dominant microorganisms in the Chinese bayberry fruits were Proteobacteria, mainly Acetobacteraceae and Burkholderiaceae. *Acetobacter* (family Acetobacteraceae) is a typical symbiotic commensal microbial species in the adult *Drosophila* midgut and is related to acetic acid production (Miladinović et al., 2015); the *Drosophila* gut commensal community *Gluconobacter* (family Acetobacteraceae) is actively shaped by host immunity and may be pathogenic (Kim et al., 2012). Burkholderiaceae is related to biodegradation, biological control and promotion of plant growth in agriculture, and has been reported that it has a good effect in inhibiting fungal diseases of fruits (Carrion et al., 2018). The application of IRPN significantly elevated the relative abundance of Burkholderiaceae in bayberry fruits. Therefore, the higher abundance of Burkholderiaceae may enhance the anti-fungal ability of fruits, to increase yield and improve fruits quality. As well as expected, the reduction of *Drosophila* infestation by either insecticides or IPNs decreased the relative abundance of *Acetobacter* and *Gluconobacter* in bayberry fruits. However, the significantly lower abundance of *Acetobacter* and *Gluconobacter* in the IRPN-treated fruits than in IPN-treated fruits suggests that not only insect avoidance but also rain avoidance inhibits the accumulation of these two bacteria in bayberry fruits. *Acetobacter* species use alcohol and glucose to produce oligosaccharides, acetic acid and other organic acids (Kulka and Walker, 1954; Walker and Pellegrino, 1959; Shin et al., 2011), and *Gluconobacter* species decompose glucose to produce gluconate (Ano et al., 2011; Wei et al., 2014). Therefore, the lower abundance of *Acetobacter* and *Gluconobacter* in fruit from IPRN-treated trees may reduce the decomposition of glucose and the production of organic acids, increasing the sweetness and reducing the acidity of the fruits.

Microbes within plant tissues have been suggested to significantly improve plant growth (Rohrbach and Schmitt, 2003), and the beneficial effects of distinct bacterial communities in the IRPN-treated bayberry trees on fruit growth and development are also suggested in this study. The predicted functional potential of bacterial communities confirmed that carbohydrate metabolism was significantly improved by treatment with IPN or IRPN compared with controls. Meanwhile, IPN or IRPN also improved galactose metabolism, starch and sucrose metabolism, ascorbate and aldarate metabolism, which may provide energy for fruit development and increase the sweetness of fruits (Chen et al., 2015). IRPN improved the metabolism of fatty acids and ketone bodies, as well as xenobiotic biodegradation, which may release energy in bayberry fruits (Schönfeld and Wojtczak, 2016), thereby reducing the consumption of sugar and increasing its accumulation in fruits, benefiting fruit growth and development. IRPN also increased amino acid metabolism in bayberry fruits, resulting in a higher accumulation of free amino acids in the fruits (Supplementary Figure S1), which are important for plant growth and development (Hernández-Verdeja and Strand, 2018). The application of IRPN increased the expression of ABC transporter genes, which may be involved in the cellular uptake and translocation of mineral elements (Köster, 2001; Neupane et al., 2019). Therefore, IRPN use is an ideal strategy to improve the fruit yield and quality of Chinese bayberry because protection against insects and rain allows the fruits to develop very specific microbial communities, which not only have a reduced abundance of pathogenic microorganisms but also have microorganisms that promote enhanced fruit growth and development, as well as accumulation of sugars and nutrients.

## Conclusion

Chinese bayberry fruits are highly sensitive to *Drosophila* and excessive rain. The present study is the first to show that a simple IRPN can be an ideal strategy for *Drosophila* control and rain-proofing, thereby significantly improving both the fruit production and quality of Chinese bayberry. The positive effects of IRPN on bayberry fruits are largely resulted from both the reduction of *Drosophila* and its unique bacterial communities shaped by *Drosophila* control and rain avoidance. IRPN significantly decreased *Acetobacter* and *Gluconobacter* in fruits to reduce sugar consumption and disease, while might increase the fruits’ functional profiles associated with carbohydrate and nitrogen metabolism, and mineral transport for improvement of fruit growth and development.

## Data Availability Statement

The original contributions presented in the study are included in the article/Supplementary Material; further inquiries can be directed to the corresponding author.

## Author Contributions

ST, QH, YW, and LL contributed to the conception and designing of the study. ST, QH, and YW provided the experimental sites and samples. HY completed the experiment and obtained the data. HY, JC, and RW performed the data and statistical analysis. HY and LL wrote the first draft of the manuscript. JC wrote sections of the manuscript. All authors contributed to manuscript revision, read and approved the submitted version.

## Funding

This work was supported by grants from the National Natural Science Foundation of China (41977130 and 31471939) and grants from the Huangyan Agricultural and Rural Bureau.

## Conflict of Interest

The authors declare that the research was conducted in the absence of any commercial or financial relationships that could be construed as a potential conflict of interest.

## Publisher’s Note

All claims expressed in this article are solely those of the authors and do not necessarily represent those of their affiliated organizations, or those of the publisher, the editors and the reviewers. Any product that may be evaluated in this article, or claim that may be made by its manufacturer, is not guaranteed or endorsed by the publisher.

## Footnotes

^1^ https://www.ncbi.nlm.nih.gov/bioproject/PRJNA723673

## Abbreviations

IPN: insect-proof net
IRPN: insect- and rain-proof net
SSC: soluble solids content
TAC: titratable acidity content
INQ: index of nutritional quality
