# Supplementary material for: An Insect- and Rain-Proof Net Raises the Production and Quality of Chinese Bayberry by Preventing Damage From Insects and Altering Bacterial Communities
Source: Front Plant Sci. 2021 Sep 16;12:732012. doi: 10.3389/fpls.2021.732012 (PMC8481806; doi:10.3389/fpls.2021.732012)
Supplement: Supplementary file 2 [file Table_1.docx]

**Table S1.** Concentration and INQ of nutrient elements in Chinese bayberry pulp under different planting environments.

| Treatments | **K** | |  | **Ca** | |  | **Mg** | |  | **Fe** | |  | **Zn** | |
| --- | --- | --- | --- | --- | --- | --- | --- | --- | --- | --- | --- | --- | --- | --- |
|  | **Conc.^*^** | **INQ^**^** |  | **Conc.** | **INQ** |  | **Conc.** | **INQ** |  | **Conc.** | **INQ** |  | **Conc.** | **INQ** |
| Control | 7.32 ± 0.27 ^a^ | 39.8 |  | 721 ± 127 ^a^ | 9.8 |  | 483 ± 12.0 ^ab^ | 15.0 |  | 51.0 ± 8.47 ^a^ | 31.7 |  | 14.1 ± 1.68 ^a^ | 11.5 |
| Insecticide | 6.36 ± 0.15 ^b^ | 28.4 |  | 280 ± 32 ^b^ | 3.1 |  | 407 ± 23.8 ^bc^ | 10.4 |  | 27.0 ± 3.05 ^b^ | 13.8 |  | 7.3 ± 0.63 ^c^ | 4.9 |
| IPN | 5.74 ± 0.19 ^c^ | 21.8 |  | 368 ± 42 ^b^ | 3.5 |  | 510 ± 31.8 ^a^ | 11.0 |  | 31.5 ± 4.38 ^b^ | 13.6 |  | 7.8 ± 0.33 ^c^ | 4.4 |
| IRPN | 4.92 ± 0.18 ^d^ | 15.0 |  | 276 ± 35 ^b^ | 2.1 |  | 377 ± 26.5 ^c^ | 6.6 |  | 23.7 ± 3.51 ^b^ | 8.3 |  | 10.2 ± 0.79 ^b^ | 4.7 |

**^*^**The units for each index of Conc. were as following: K (g/kg), Ca (mg/kg), Mg (mg/kg), Fe (mg/kg), Zn (mg/kg). The mean and standard deviation (n = 3) were calculated for three replicates. The values in a column with different superscript letters are significantly different (P<0.05). IPN: insect-proof net, IRPN: insect-and rain-proof net, Conc.: Concentration. The letters a, b, c and d indicate significant difference.

**^**^**INQ: Index of nutritional quality.

**Figure S1.** The influence of different planting environments on the concentrations (mg/g) (A) and accumulation (mg) (B) of free amino acids.
